# Neural Correlates of Sexual Cue Reactivity in Individuals with and without Compulsive Sexual Behaviours

**DOI:** 10.1371/journal.pone.0102419

**Published:** 2014-07-11

**Authors:** Valerie Voon, Thomas B. Mole, Paula Banca, Laura Porter, Laurel Morris, Simon Mitchell, Tatyana R. Lapa, Judy Karr, Neil A. Harrison, Marc N. Potenza, Michael Irvine

**Affiliations:** 1 Department of Psychiatry, Addenbrooke's Hospital, University of Cambridge, Cambridge, United Kingdom; 2 Behavioural and Clinical Neurosciences Institute, University of Cambridge, Cambridge, United Kingdom; 3 Cambridgeshire and Peterborough Foundation Trust, Cambridge, United Kingdom; 4 British Association for Counselling and Psychotherapy, London, United Kingdom; 5 Department of Psychiatry, Brighton and Sussex Medical School, Brighton, United Kingdom; 6 Departments of Psychiatry, Neurobiology and Child Study Center, Yale University, New Haven, Connecticut, United States of America; INSERM/CNRS, France

## Abstract

Although compulsive sexual behaviour (CSB) has been conceptualized as a “behavioural” addiction and common or overlapping neural circuits may govern the processing of natural and drug rewards, little is known regarding the responses to sexually explicit materials in individuals with and without CSB. Here, the processing of cues of varying sexual content was assessed in individuals with and without CSB, focusing on neural regions identified in prior studies of drug-cue reactivity. 19 CSB subjects and 19 healthy volunteers were assessed using functional MRI comparing sexually explicit videos with non-sexual exciting videos. Ratings of sexual desire and liking were obtained. Relative to healthy volunteers, CSB subjects had greater desire but similar liking scores in response to the sexually explicit videos. Exposure to sexually explicit cues in CSB compared to non-CSB subjects was associated with activation of the dorsal anterior cingulate, ventral striatum and amygdala. Functional connectivity of the dorsal anterior cingulate-ventral striatum-amygdala network was associated with subjective sexual desire (but not liking) to a greater degree in CSB relative to non-CSB subjects. The dissociation between desire or wanting and liking is consistent with theories of incentive motivation underlying CSB as in drug addictions. Neural differences in the processing of sexual-cue reactivity were identified in CSB subjects in regions previously implicated in drug-cue reactivity studies. The greater engagement of corticostriatal limbic circuitry in CSB following exposure to sexual cues suggests neural mechanisms underlying CSB and potential biological targets for interventions.

## Introduction

Excessive or problematic engagement in sex, which has been termed compulsive sexual behaviour (CSB), hypersexuality disorder or sexual addiction, is a relatively common clinical entity that may carry significant mental and physical health consequences [1]. Although precise estimates are unknown as many major psychiatric epidemiological studies do not include measures of CSB, existing data suggest that rates for CSB may range from 2 to 4% in community and college-based young adults with similar rates in psychiatric inpatients [2]–[4], although higher and lower rates have been reported depending on how CSB is defined [5]. A complicating factor in determining the precise prevalence and impact of CSB involves the lack of a formal definition for the disorder. Although criteria for hypersexual disorder were proposed for DSM-5 [6], the disorder was not included in DSM-5. However, as CSB may be associated with significant distress, feelings of shame and psychosocial dysfunction, it warrants direct examination.

How best to conceptualize CSB has been debated, with rationales proposed for considering the condition as an impulse-control disorder or a non-substance or “behavioural” addiction [7]. Based on existing data, pathological gambling (or gambling disorder) was recently reclassified in DSM-5 together with substance-use disorders as a behavioural addiction [8]. However, other disorders (e.g., those relating to excessive engagement in Internet use, video-gaming or sex) were not included in the main section of the DSM-5, in part due to limited data on the conditions [9]. Thus, an improved understanding of CSB and how it might show similarities to or differences from substance-use disorders may help with classification efforts and the development of more effective prevention and treatment efforts. Given similarities between substance-use, gambling and hypersexual disorders (e.g., in impaired control over pleasurable or rewarding behaviours), the investigation of elements salient to addictions (e.g., cue reactivity) warrant direct investigation in CSB.

Cue reactivity relates importantly to clinically relevant aspects of substance-use disorders. For example, heightened cue reactivity is associated with relapse [10], [11]. A recent quantitative meta-analysis of studies in cue reactivity across substances of misuse including alcohol, nicotine and cocaine demonstrated overlapping activity to drug cues in the ventral striatum, dorsal anterior cingulate (dACC) and amygdala, with overlapping activity to self-reported cue-induced craving in dACC, pallidum and ventral striatum [11]. However, the extent to which these regions may show differential sexual-cue reactivity in individuals with and without CSB has not been studied.

Different models have been proposed to explain addictive behaviours, with one model positing that in addictions, “wanting” becomes dissociated from “liking” as one becomes addicted [12]. However, the extent to which liking and wanting relate to sexual-cue reactivity and its neural correlates in CSB has not been systematically examined, and findings from such studies may provide data to help guide the most appropriate classification of CSB and identify neural targets for treatment development.

Multiple studies have previously focused on sexual cues in healthy volunteers identifying regions including the hypothalamus, thalamus, amygdala, anterior cingulate cortex, anterior insula, inferior frontal cortex, fusiform gyrus, precentral gyrus, parietal cortex and middle occipital cortex [13]–[19]. These regions are implicated in physiological and emotional arousal, attention and particularly visuospatial attention, and motivation. Using measures of penile tumescence, the striatum, anterior cingulate, insula, amygdala, occipital cortex, sensorimotor cortex and hypothalamus have been shown to play a role in penile erection [15], [20]. Gender-related differences have been reported with males having greater amygdala and hypothalamic activity to sexual stimuli relative to females, and these differences may reflect appetitive states [21]. A meta-analysis identified a common brain network to monetary, erotic and food outcomes including the ventromedial prefrontal cortex, ventral striatum, amygdala, anterior insula and mediodorsal thalamus [22]. Food and erotic rewards were associated particularly with anterior insular activity and erotic rewards more specifically with amygdala activity. A recent study has also shown that longer duration of use of online explicit materials in healthy males correlates with lower left putaminal activity and lower right caudate volumes to brief still sexual images [23].

Neurophysiological studies focusing on CSB in the general population rather than healthy volunteers are comparatively more limited. A diffusion MRI study focusing on a small group of non-paraphilic CSB subjects (N = 8) compared to healthy volunteers (N = 8) showed lower mean diffusivity in superior frontal regions [24]. Subjects were recruited from a treatment program with 7 of 8 subjects having a history of alcohol use disorders, 4 of 8 with a history of other substance abuse or dependence and 1 of 8 with a history of obsessive compulsive disorder. In a study focusing on 52 male and female CSB subjects with problems regulating online viewing of sexual images recruited from online advertisements, exposure to static sexual images compared to neutral images was associated with elevated amplitudes of the P300 response, implicated in attentional control [25]. As this measure correlated with dyadic sexual desire but not sexual compulsivity measures, the authors suggested the P300 amplitude mediated sexual desire rather than compulsive behaviours. Hypersexuality has been reported in the context of neurological disorders and their associated medications. Compulsive hypersexuality, occurring in 3–4% of Parkinson's disease patients and related to dopaminergic medications [26], [27], has also been studied using imaging modalities. A case report using technetium-99 m-ethyl cysteinate dimer SPECT showed relatively increased blood flow in mesial temporal regions in the CSB patient [28]. A larger study focusing on Parkinson's disease patients with hypersexuality showed greater functional MRI Blood Oxygen Level Dependent activity to sexual picture cues that correlated with enhanced sexual desire [29], which the authors suggested might reflect incentive-motivation theories of addiction. A voxel-based-morphometry study of hypersexuality commonly reported in behavioural variant frontotemporal dementia, a disease which affects ventromedial frontal and anterior temporal regions, showed greater atrophy in the right ventral putamen and pallidum in association with reward-seeking scores [30]. Of note, in this sample, hypersexuality was reported in 17% with other reward seeking behaviours including overeating in 78% and new or increased alcohol or drug use in 26% of individuals in this study. In this current study, we focus on CSB subjects in the general population.

Here we assessed cue reactivity comparing sexually explicit video cues with non-sexual exciting stimuli (such as videos of sporting activities) and assessed scores of sexual desire or wanting and liking in subjects with and without CSB. We hypothesized that individuals with CSB as compared to those without would show greater desire (wanting) but not liking (similar across groups) in response to sexually explicit but not to non-sexually exciting cues. Although a range of regions have been implicated in response to sexual cues in healthy volunteers, as we were studying patients with CSB, we hypothesized that there would be greater activation to sexually explicit as compared to non-sexual exciting cues in regions implicated in drug cue reactivity studies including the ventral striatum, dACC and amygdala. We further hypothesized that these regional activations would be functionally linked across groups but more strongly in individuals with CSB as compared to those without, and that sexual desire (wanting) would be more strongly linked to activity within these regions in individuals with CSB as compared to those without. Given developmental changes in motivational systems underlying risky behaviours [31], we also explored relationships with age.

## Methods

CSB subjects were recruited via Internet-based advertisements and from referrals from therapists. Healthy volunteers were recruited from community-based advertisements in the East Anglia area. For the CSB group, screening was conducted using the Internet Sex Screening Test (ISST) [32] and an extensive investigator-designed questionnaire on details including age of onset, frequency, duration, attempts to control use, abstinence, patterns of use, treatment and negative consequences. CSB subjects underwent a face-to-face interview with a psychiatrist to confirm they fulfilled diagnostic criteria for CSB [6], [33], [34] (Table S1 in File S1) focusing on compulsive use of online sexually explicit material. All participants met proposed diagnostic criteria for Hypersexual Disorder [6], [33] and criteria for sexual addiction [34] (Table S1 in File S1).

By design and given the nature of the cues, all CSB subjects and healthy volunteers were male and heterosexual. Male healthy volunteers were age-matched (+/−5 years of age) with CSB subjects. An additional 25 age-matched male heterosexual healthy volunteers underwent the video ratings outside of the scanner to ensure adequacy of the subjective responses to the videos as assessed by subjective responses. Exclusionary criteria included being under 18 years of age, having a history of substance-use disorders, being a current regular user of illicit substances (including cannabis), and having a serious psychiatric disorder, including current moderate-severe major depression (Beck Depression Inventory >20) or obsessive-compulsive disorder, or history of bipolar disorder or schizophrenia (Mini International Neuropsychiatric Inventory) [35]. Other compulsive or behavioural addictions were also exclusions. Subjects were assessed by a psychiatrist regarding problematic use of online gaming or social media, pathological gambling or compulsive shopping, childhood or adult attention deficit hyperactivity disorder, and binge-eating disorder diagnosis. Subjects were also screened for compatibility with the MRI environment.

Subjects completed the UPPS-P Impulsive Behaviour Scale [36] to assess impulsivity, Beck Depression Inventory [37] and State Trait Anxiety Inventory [38] to assess depression and anxiety, respectively, Obsessive-Compulsive Inventory-R to assess obsessive-compulsive features and the Alcohol-Use Disorders Identification Test (AUDIT) [39]. General Internet use was assessed using the Young's Internet Addiction Test (YIAT) [40] and the Compulsive Internet Use Scale (CIUS) [41]. The National Adult Reading Test [42] was used to obtain an index of IQ. A modified version of the Arizona Sexual Experiences Scale (ASES) [43] was used with one version relevant to intimate relationships and another version relevant to online sexually explicit material.

Subject characteristics are reported in Table S1 in File S1. CSB subjects had higher depression and anxiety scores (Table S2 in File S1) but no current diagnoses of major depression. Two of 19 CSB subjects were taking antidepressants or had comorbid generalized anxiety disorder and social phobia (N = 2) or social phobia (N = 1) or a childhood history of ADHD (N = 1). One CSB subject and 1 healthy volunteer used cannabis intermittently.

Written informed consent was obtained, and the study was approved by the University of Cambridge Research Ethics Committee. Subjects were paid for their participation.

### Behavioural statistics

Subject characteristics and questionnaire scores were compared using independent t-tests or Chi-square tests. Multivariate analyses were used for the ASES scores. For the ratings of sexual desire or liking, mixed-measures ANOVA were used to compare the explicit versus erotic ratings with group (CSB, non-CSB) as a between-subjects measure, video type (explicit or erotic cues), and subjective rating (desire or liking) as within-subjects measures.

### Neuroimaging

In the imaging task, subjects viewed video clips presented in a counter-balanced fashion from one of 5 conditions: explicit sexual, erotic, non-sexual exciting, money and neutral. The videos were shown for 9 seconds, followed by a question if the video was indoors or outdoors. Subjects responded using a 2-button key-pad with their second and third digits of their right hand to ensure they were paying attention. The question occurred during a jittered inter-trial interval of 2000 to 4000 milliseconds. Explicit videos showed consensual sexual interactions between a man and a woman obtained from videos downloaded from the Internet with licenses obtained where necessary. Examples of erotic videos included a dressed woman dancing erotically or a scene of a woman brushing her thigh. Non-sexual exciting videos displayed sporting videos similar in nature to highly arousing images from the International Affective Picture System such as skiing, sky-diving, rock-climbing, or motorcycle-riding. Money videos showed images of coins or paper money being paid, falling or scattered. Neutral videos showed scenes of landscapes. The conditions were randomized with eight trials per condition shown for a total of 40 video clips. Five different videos per condition were shown for a total of 25 different video clips.

In the video-rating task outside of the scanner, subjects watched the same videos and completed a continuous rating scale for sexual desire and liking. Subjects were asked the following questions on 2 separate slides: ‘How much did this increase your sexual desire?’ and ‘How much did you like this video?’ and indicated an answer using a mouse along a line anchored from ‘Very little’ to ‘Very much’. An additional 25 male healthy volunteers were tested on the video-rating task. Subjects were asked if they have previously viewed the videos prior to the study. All tasks were coded using E-Prime 2.0 software.

### Data acquisition and processing

The acquisition parameters of the fMRI study are described in File S1. The 9-second video clips and inter-trial intervals were modelled as box-car functions convolved with hemodynamic response functions. Analyses were conducted using general linear modelling. The video conditions were compared using ANOVA with group (CSB, non-CSB) as a between-subjects factor and condition (video type) as a within-subjects factor. The main effects of group across all conditions were first compared. The effects of condition were compared individually contrasting explicit, erotic and money conditions with the exciting condition. The exciting sports videos were used as a control for the explicit and erotic conditions as they both involved moving individuals in the videos. Activations above whole-brain family-wise error (FWE) corrected P<0.05 were considered significant in the main effects comparisons. Group-by-condition (e.g. CSB(explicit – exciting) – Healthy volunteer(explicit – exciting)) interactions focusing on *a priori* hypothesized regions of interest were conducted if the contrast of condition (e.g. explicit – exciting) identified regions significant at the whole-brain FWE P<0.05 level. Age and depression scores were used as covariates. Variables including subjective measures of sexual desire and liking responses to the video cues, scores on the Young Internet Addiction Test, and days abstinent were included in models as covariates of interest. The covariate of age was also investigated, controlling for depression and subjective desire, across groups and using explicit masking.

The ventral striatum, amygdala and dorsal cingulate were hypothesized regions of interest. For these three regions with strong *a priori* hypotheses, we combined the ROIs using a small-volume-correction (SVC) with Family-Wise-Error correction at p<0.05 considered significant. Given findings linking subjective ratings of desire to dorsal anterior cingulate activation, psychophysiological interaction analysis was conducted with dorsal cingulate as the seed region (coordinates x y z = 0 8 38 mm, radius = 10 mm) contrasting explicit – exciting videos. Given the potential involvement of mesolimbic and mesocortical circuitry, activity in the substantia nigra was also assessed on an exploratory level. The ventral striatal anatomical region of interest (ROI), previously used in other studies [44], had been hand drawn in MRIcro following the definition of ventral striatum by Martinez et al. [45]. The ROIs for cingulate and amygdala were obtained from aal templates in WFUPickAtlas SPM Toolbox [46]. Two different templates for the substantia nigra ROI were used including the WFUPickAtlas template and a hand-drawn ROI in MRIcro using magnetization transfer sequences from 17 healthy volunteers. All imaging data were pre-processed and analysed using SPM 8 (Wellcome Trust Centre for NeuroImaging, London, UK).

## Results

### Characteristics

Nineteen heterosexual men with CSB (age 25.61 (SD 4.77) years) and 19 age-matched (age 23.17 (SD 5.38) years) heterosexual male healthy volunteers without CSB were studied (Table S2 in File S1). An additional 25 similarly aged (25.33 (SD 5.94) years) male heterosexual healthy volunteers rated the videos. CSB subjects reported that as a result of excessive use of sexually explicit materials, they had lost jobs due to use at work (N = 2), damaged intimate relationships or negatively influenced other social activities (N = 16), experienced diminished libido or erectile function specifically in physical relationships with women (although not in relationship to the sexually explicit material) (N = 11), used escorts excessively (N = 3), experienced suicidal ideation (N = 2) and using large amounts of money (N = 3; from £7000 to £15000). Ten subjects either had or were in counselling for their behaviours. All subjects reported masturbation along with the viewing of online sexually explicit material. Subjects also reported use of escort services (N = 4) and cybersex (N = 5). On an adapted version of the Arizona Sexual Experiences Scale [43], CSB subjects compared to healthy volunteers had significantly more difficulty with sexual arousal and experienced more erectile difficulties in intimate sexual relationships but not to sexually explicit material (Table S3 in File S1).

Compared to healthy volunteers, CSB subjects first viewed online sexually explicit materials at an earlier age (HV: 17.15 (SD 4.74); CSB: 13.89 (SD 2.22) in years) relative to the age of onset for Internet use in general (HV: 12.94 (SD 2.65); CSB: 12.00 (SD 2.45) in years) (group-by-onset interaction: F(1,36) = 4.13, p = 0.048). CSB subjects had greater Internet use relative to healthy volunteers (Table S3 in File S1). Importantly, CSB subjects reported using the Internet for viewing online sexually explicit material for 25.49% of total online use (for an average 8.72 (SD 3.56) years) compared to 4.49% in healthy volunteers (t = 5.311, p<0.0001) (CSB vs. HV: sexually explicit material use: 13.21 (SD 9.85) vs. 1.75 (SD 3.36) hours per week; total internet use: 37.03 (SD 17.65) vs. 26.10 (18.40) hours per week).

### Cue reactivity

Subjective ratings of desire and liking of videos were dissociated in which there was a group-by-rating-type-by-video-type interaction (F(1,30) = 4.794, p = 0.037): desire ratings to explicit videos were greater in CSB compared to healthy volunteers (F = 5.088, p = 0.032) but not to erotic cues (F = 0.448, p = 0.509), whereas liking ratings to erotic cues were greater in CSB compared to healthy volunteers (F = 4.351, p = 0.047) but not to explicit cues (F = 3.332, p = 0.079). The desire and liking scores to explicit cues were significantly correlated (HV: R^2^ = 0.696, p<0.0001; CSB: R^2^ = 0.363, p = 0.017) although the linear regression was not significantly different between groups (F = 2.513, p = 0.121). There were also no differences in the video-rating scores for desire and liking for each condition between the scanned healthy volunteers and an additional 25 healthy volunteers suggesting the subjective ratings to the videos were representative (p's>0.05). All subjects reported they had not previously seen the videos prior to the study.

### Imaging analyses

**Figure 1:**
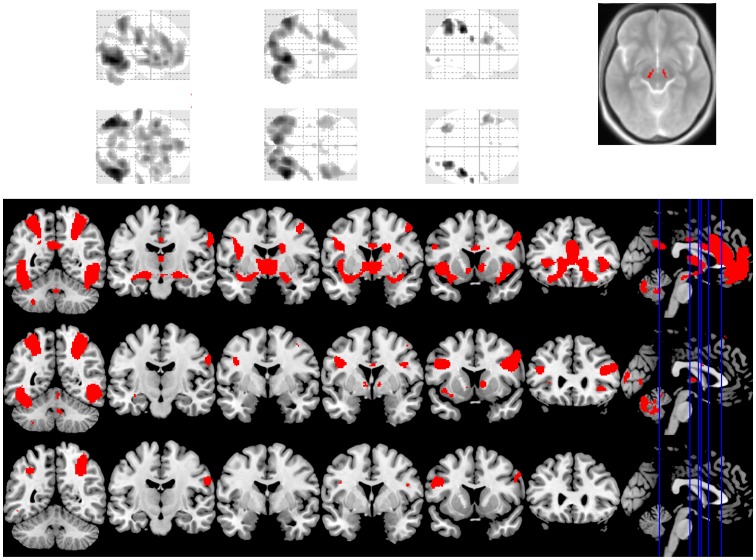
Condition contrasts. The glass brains and coronal images show the effects across groups of the following contrasts: explicit – exciting (left, top row), erotic – exciting (middle, middle row) and money – exciting (right, bottom row). The images are shown at whole-brain FWE-corrected P<0.05. The axial view (top right) shows the contrast across groups of explicit – exciting videos focusing on the substantia nigra. The image is shown with a substantia nigra region of interest mask overlaid on a magnetization transfer sequence.

No between-group main-effect brain activation differences survived whole-brain correction. The contrast of explicit – exciting videos across subject groups identified activation of the ventral striatum, dACC and amygdala at the whole-brain-corrected FWE p<0.05 level (Figure 1, Tables S4 and S5 in File S1). The contrast also identified bilateral activation of the hypothalamus and substantia nigra (whole-brain-corrected FWE p<0.05), regions implicated in sexual arousal and dopaminergic function, respectively [13], [22]. The contrasts of explicit – exciting and erotic – exciting both identified activity in bilateral occipito-temporal regions, parietal and inferior frontal cortices and right caudate (whole-brain-corrected FWE p<0.05) (Table S4 in File S1). However, the contrast of erotic – exciting did not identify *a priori* hypothesized regions. Similarly, the money – exciting contrast identified bilateral parietal and inferior frontal cortices (whole-brain-corrected FWE p<0.05) but not the *a priori* hypothesized regions.

**Figure 2:**
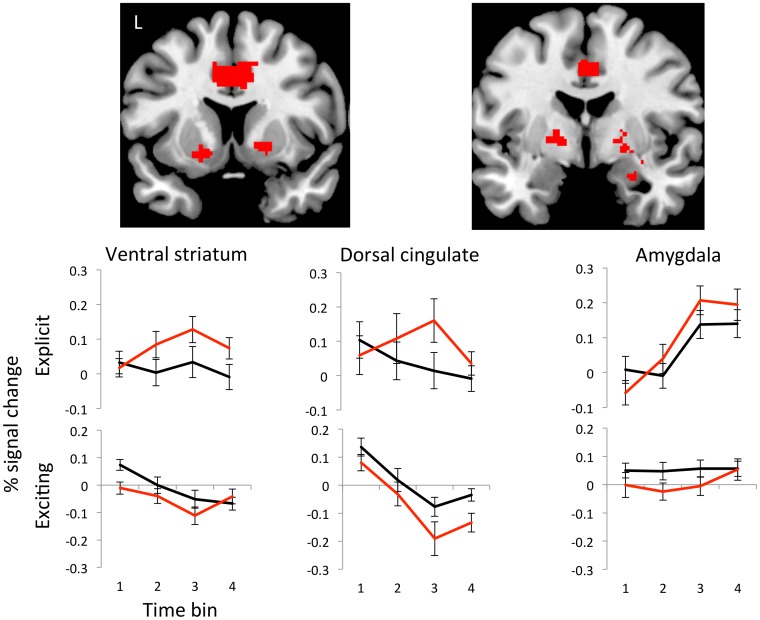
Explicit versus exciting cues. The coronal views represent the group-byvideo-type interaction of subjects with compulsive sexual behaviour (CSB)>healthy volunteers (HV) contrasting explicit>exciting cues. The images are shown as regions of interest at P<0.005. The time course analyses represent the % signal change to explicit videos (top) and exciting videos (bottom) with CSB subjects in red and healthy volunteers in black. Error bars represent SEM.

We next examined between-group differences in the explicit – exciting contrast which had shown a significant effect across groups in our hypothesized regions. CSB subjects demonstrated greater activity in the right ventral striatum (peak voxel x y z in mm = 18 2 −2, Z = 3.47, FWE p = 0.032), dACC (0 8 38, Z = 3.88, FWE p = 0.020) and right amygdala (32 −8 −12, Z = 3.38, FWE p = 0.018) (Figure 2). Given a role for dopaminergic circuitry in cue reactivity, we also explored activity in the substantia nigra. CSB subjects had greater activity in the right substantia nigra (10 −18 −10, Z = 3.01, FWE p = 0.045) in the explicit – exciting contrast. A sub-analysis excluding the two subjects who were on antidepressants did not change the significant findings.

**Figure 3:**
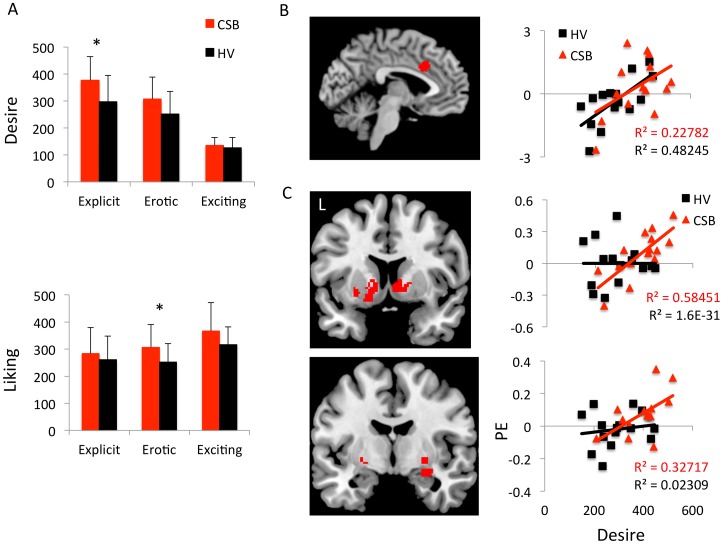
Sexual desire. A. Subjective desire and liking scores to video types in subjects with compulsive sexual behaviours (CSB) and healthy volunteer (HV) participants. There was a significant group-by-video-type-by-desire/liking interaction. Error bars represent SEM. *p<0.05. B. Desire covariate for explicit videos in both CSB and HV subjects with the corresponding regression analysis graph for dorsal cingulate parameter estimates (P.E.) and desire scores. C. Psychophysiological interaction analysis with desire covariate for explicit-exciting contrast with dorsal cingulate seed. The coronal images and graphs show CSB subjects with an HV exclusive mask and corresponding regression analyses for ventral striatum and amygdala parameter estimates and desire scores. The images are shown as regions of interest at P<0.005.

To examine the relationship between neural response to cues and ratings of desire and liking, we conducted covariate analyses involving brain responses to the explicit cues. In both groups, ratings of subjective sexual desire were positively correlated with dACC activity (−4 18 32, Z = 3.51, p = 0.038), with no differences between groups (Figure 3). There were no neural correlations with subjective liking.

**Figure 4:**
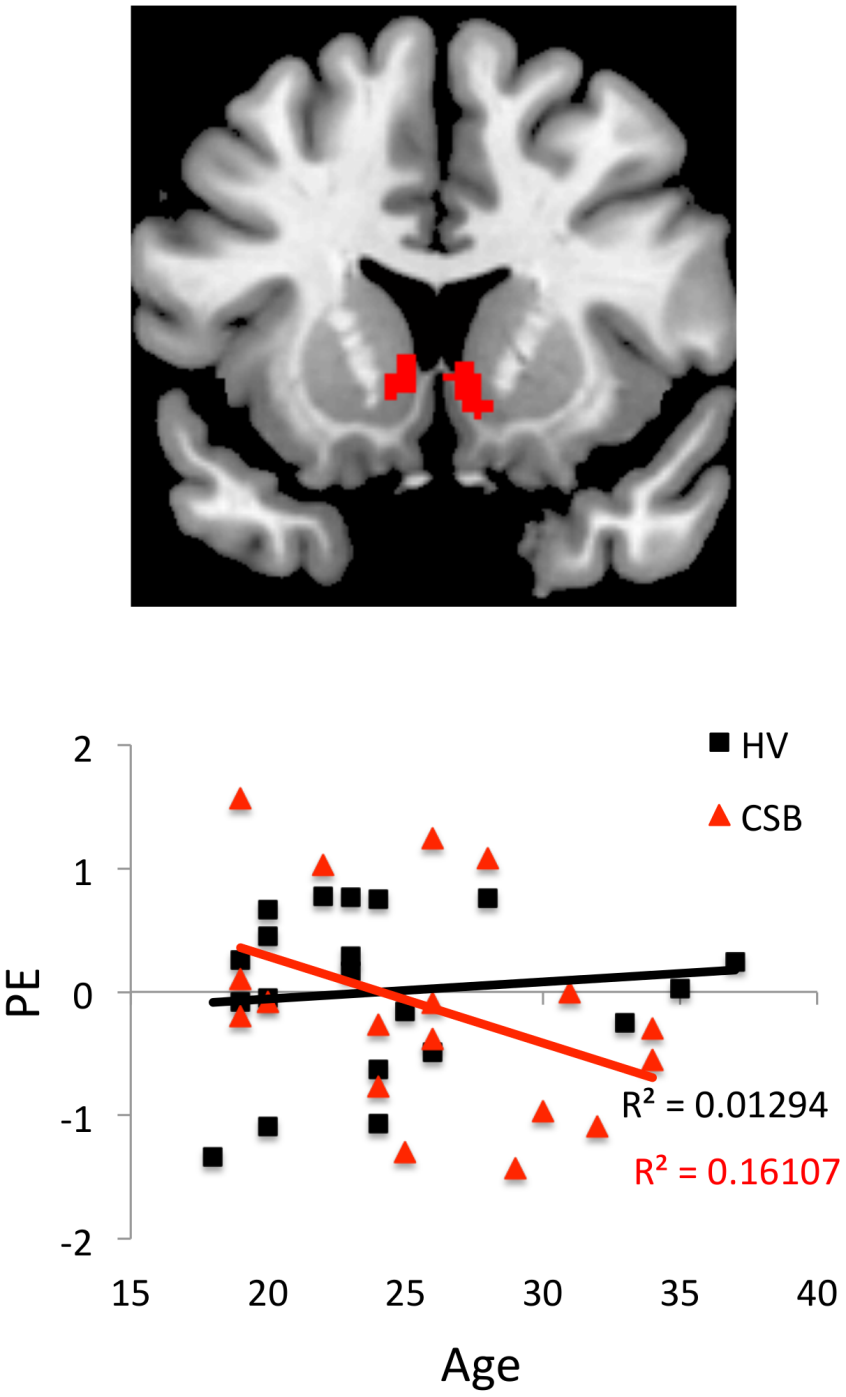
Age. The coronal view shows the age covariate for explicit videos in subjects with Compulsive Sexual Behaviours (CSB) with a healthy volunteer (HV) exclusive mask. The graph shows the corresponding regression analysis for the ventral striatal parameter estimate (PE) and age in years. The image is shown as a region of interest at P<0.005.

On an exploratory level, neural activity was investigated as a function of age. Age across all subjects was negatively correlated with activity in the right ventral striatum (right: 8 20 −8, Z = 3.13, FWE p = 0.022) and dACC (2 20 40, Z = 3.88, FWE p = 0.045). Greater activity as a function of age was observed in the CSB group as compared to healthy volunteers in bilateral ventral striatum (right: 4 18 −2, Z = 3.31, FWE p = 0.013; left −8 −18 −2, Z = 3.01, FWE p = 0.034) (Figure 4).

Given the association between ratings of subjective sexual desire dACC activity, a psychophysiological interaction analysis using the dACC as a seed was conducted comparing explicit – exciting cues. Across both groups, there was increased functional connectivity of the dACC with the right ventral striatum (8 20 −4, Z = 3.14, FWE p = 0.029) and right amygdala (12 0 −18, Z = 3.38, FWE p = 0.009). There were no between-group differences in functional connectivity. When subjective desire scores were assessed as a covariate, there was a positive correlation between desire scores and greater functional connectivity in CSB subjects between the dACC and right ventral striatum (12 2 −2, Z = 3.51, FWE p = 0.041) and right amygdala (30 −2 −12, Z = 3.15, FWE p = 0.048) (Figure 3) and, on an exploratory level, left substantia nigra (−14 −20 −8, Z = 3.10, FWE p = 0.048) compared to healthy volunteers. There were no significant findings relating to liking measures.

## Discussion

In this study of sexually explicit, erotic and non-sexual cues, individuals with CSB and those without showed similarities and differences with respect to patterns of neural responsivity and relationships between subjective and neural responses. Sexual desire or wanting of the explicit sexual cues was linked to a dACC-ventral striatal-amygdala functional network evident across both groups and more strongly activated and linked to sexual desire in the CSB group. Sexual desire or subjective measures of wanting appeared dissociated from liking, in line with incentive-salience theories of addiction [12] in which there exists enhanced wanting but not liking of salient rewards. We further observed a role for age in which younger age, particularly in the CSB group, was associated with greater activity in the ventral striatum.

Compared to healthy volunteers, CSB subjects had greater subjective sexual desire or wanting to explicit cues and had greater liking scores to erotic cues, thus demonstrating a dissociation between wanting and liking. CSB subjects also had greater impairments of sexual arousal and erectile difficulties in intimate relationships but not with sexually explicit materials highlighting that the enhanced desire scores were specific to the explicit cues and not generalized heightened sexual desire. In CSB subjects as compared to healthy volunteers, higher scores of sexual desire to explicit cues were associated with greater dACC activity and enhanced functional connectivity between the dACC, ventral striatum and amgydala (as described below), suggesting a network involved in the processing of subjective wanting related to sexual cues. A previous study of compulsive hypersexuality related to dopamine agonists in Parkinson's disease, which can include behaviours such as compulsive use of sexually explicit materials, demonstrated greater neural activity to sexual picture cues that correlated with enhanced sexual desire [29]. Our findings focusing on CSB in the general population similarly dovetail with incentive motivation theories emphasizing aberrant wanting or motivation towards the drug or sexual cue, but not of ‘liking’ or hedonic tone [12].

Drug-cue-reactivity and craving studies of nicotine, cocaine and alcohol implicate networks including the ventral striatum, dACC and amygdala [13]. In the current study, these regions were activated during viewing of sexually explicit materials across the groups with and without CSB. The observation of stronger activations of these regions in CSB versus healthy volunteer participants is similar to findings observed for substance cues in substance addictions, suggesting neurobiological similarities across the disorders.

In the current study in response to sexually explicit cues, sexual desire was associated with greater dACC activity, and greater dACC-ventral striatal-amygdala functional network activity was related to enhanced desire to a greater extent in the CSB subjects than in the healthy volunteer subjects. CSB subjects also demonstrated greater substantia nigra activity compared to healthy volunteers, thus possibly linking the findings to dopaminergic activity. In humans and non-human primates, the dACC is an important target of dopaminergic projections from the substantia nigra and ventral tegmental area [47], tracking salience and prediction error signals. The dACC sends anatomical projections to the ventral and dorsomedial striatum, implicated in the representation of value and reward signals and motivation and has reciprocal connections to the lateral basal nucleus of the amygdala thus receiving information on emotionally salient events [48], [49]. The region also has multiple connections with cortical regions including premotor, primary motor and fronto-parietal cortices and is well-localized to influence action selection. The dACC is implicated in the processing of pain, negative stimuli and cognitive control [48], with recent studies highlighting the role of the dACC in prediction error signalling and reward expectation [50], [51], particularly to guide action-reward learning [52], [53]. Our functional connectivity findings dovetail with a role for a network converging on the dACC in the processing of sexual rewards and in sexual-cue-related reactivity and its relationship to desire as a motivational signal.

Our findings suggest dACC activity reflects the role of sexual desire, which may have similarities to a study on the P300 in CSB subjects correlating with desire [25]. We show differences between the CSB group and healthy volunteers whereas this previous study did not have a control group. The comparison of this current study with previous publications in CSB focusing on diffusion MRI and the P300 is difficult given methodological differences. Studies of the P300, an event related potential used to study attentional bias in substance use disorders, show elevated measures with respect to use of nicotine [54], alcohol [55], and opiates [56], with measures often correlating with craving indices. The P300 is also commonly studied in substance-use disorders using oddball tasks in which low-probability targets are frequently mixed with high-probability non-targets. A meta-analysis showed that substance-use-disordered subjects and their unaffected family members had decreased P300 amplitude compared to healthy volunteers [57]. These findings suggest substance-use disorders may be characterized by impaired allocation of attentional resources to task-relevant cognitive information (non-drug targets) with enhanced attentional bias to drug cues. The decrease in P300 amplitude may also be an endophenotypic marker for substance-use disorders. Studies of event-related potentials focusing on motivation relevance of cocaine and heroin cues further report abnormalities in the late components of the ERP (>300 milliseconds; late positive potential, LPP) in frontal regions, which may also reflect craving and attention allocation [58]–[60]. The LPP is believed to reflect both early attentional capture (400 to 1000 msec) and later sustained processing of motivationally significant stimuli. Subjects with cocaine use disorder had elevated early LPP measures compared to healthy volunteers suggesting a role for early attentional capture of motivated attention along with attenuated responses to pleasant emotional stimuli. However, the late LPP measures were not significantly different from those in healthy volunteers [61]. The generators of the P300 event-related potential for target-related responses is believed to be the parietal cortex and cingulate [62]. Thus, both dACC activity in the present CSB study and P300 activity reported in a previous CSB study may reflect similar underlying processes of attentional capture. Similarly, both studies show a correlation between these measures with enhanced desire. Here we suggest that dACC activity correlates with desire, which may reflect an index of craving, but does not correlate with liking suggestive of on an incentive-salience model of addictions.

The current findings suggest age-related influences on the processing of sexual cues. Maturation of fronto-cortical grey matter involved in executive control persists in adolescence into the mid-20 s [63]. Enhanced risk taking in adolescents is may reflect earlier development of limbic incentive motivation and reward circuitry relative to more delayed development of frontal executive control systems involved in monitoring or inhibiting behaviours [31], [64], [65]. For instance, adolescents have demonstrated greater ventral striatal activity relative to prefrontal cortical activity during reward processing compared to adults [65]. Here we observe that across subjects, young age is associated with greater ventral striatal activity to sexually explicit cues. This effect in ventral striatal activity appears particularly robust in CSB subjects, suggesting a potential modulatory role of age on responses to sexual cues in general and in CSB specifically.

In keeping with the literature on brain activity in healthy volunteers to explicit sexual stimuli activated regions, we show a similar network including the occipito-temporal and parietal cortices, insula, cingulate and orbitofrontal and inferior frontal cortices, pre-central gyrus, caudate, ventral striatum, pallidum, amygdala, substantia nigra and hypothalamus [13]–[19]. Longer duration of use of online explicit materials in healthy males has been shown to correlate with lower left putaminal activity to brief still explicit images suggesting a potential role of desensitization [23]. In contrast, this current study focuses on a pathological group with CSB characterized by difficulty with controlling use associated with negative consequences. Furthermore, this current study uses video clips as compared to brief still images. In healthy volunteers, viewing of erotic still images compared to video clips has a more limited activation pattern including hippocampus, amygdala and posterior temporal and parietal cortices [20] suggesting possible neural differences between the brief still images and the longer videos used in this current study. Furthermore, disorders of addiction such as cocaine use disorders have also been shown to be associated with enhanced attentional bias whereas recreational cocaine users have not been shown to have enhanced attentional bias [66] suggesting potential differences between recreational versus dependent users. As such, differences between studies may reflect differences in the population or task. Our study suggests that the brain responses to explicit online materials may differ between subjects with CSB as compared to healthy individuals who may be heavy users of explicit online materials but without the loss of control or association with negative consequences.

The current study has multiple limitations. First, the study involved only heterosexual male subjects, and future studies should examine individuals of various sexual orientations and females, particularly as girls with mental health concerns may exhibit high rates of CSB [67]. Second, although CSB subjects in the study met the provisional diagnostic criteria and demonstrated functional impairment relating to sex using multiple validated scales, there currently exist no formal diagnostic criteria for CSB and thus this represents a limitation for understanding the findings and placing them within the larger literature. Third, given the cross-sectional nature of the study, inferences about causality cannot be made. Future studies should examine the extent to which neural activation to sexual cues may represent potential risk factors indicating enhanced vulnerability or whether repeated exposure, possibly influenced by younger age and greater exposure to sexually explicit material, might lead to the neural patterns observed in CSB. Further studies of a prospective nature or those focusing on unaffected family members are warranted. The restricted age range in the study may also limit possible findings. Fourth, our study focused predominantly on compulsive use of online materials with associated masturbation and less frequently use of cybersex or use of escort services. As these subjects were recruited from both online advertisements and treatment settings, whether they fully represent subjects in treatment settings is less clear. A study of 207 treatment-seeking CSB subjects used in a DSM-5 field trial for the diagnosis of hypersexual disorder similarly noted the most frequent behaviours being pornography use (81.1%), masturbation (78.3%), cybersex (18.1%) and sex with consenting adults (44.9%) [33] suggesting similarities between our population and this reported subject population. However, studies focusing on a treatment seeking population may reflect greater severity of symptoms. We used a region of interest analysis rather than a more whole brain approach. Thus, the small sample and lack of a whole brain corrected approach is a limitation. However, given our strong *a priori* hypotheses based on available meta-analytic data from cue reactivity studies, we felt a region of interest analysis family wise error corrected for multiple comparisons, an approach commonly used in imaging studies [68], was a reasonable approach.

The current and extant findings suggest that a common network exists for sexual-cue reactivity and drug-cue reactivity in groups with CSB and drug addictions, respectively. These findings suggest overlaps in networks underlying disorders of pathological consumption of drugs and natural rewards. While this study may suggest overlaps with substance-use disorders, further clinical studies are required to determine whether CSB should be categorized as an impulse-control disorder, within an obsessive-compulsive spectrum or as a behavioural addiction. Large multi-centre epidemiological studies with long-term follow-up are required to assess the frequency of CSB and its long-term outcomes. Epidemiological studies on the relationship between CSB and disorders of impulsivity, compulsivity and addictions are required. Similarly, more extensive comparisons on neurocognitive and neurophysiological profiles across disorders would be helpful in further understanding the physiology and neural networks underlying these disorders. We emphasize also that these findings are relevant particularly to the subgroup of individuals who develop difficulties with compulsive use of online sexually explicit materials and likely do not reflect on the wider population who use such materials in non-harmful manners. The findings indicate an influence of age on enhanced limbic reactivity to sexual rewards, particularly in the CSB group. Given the recent increases in Internet use, including among young individuals, and the ready access to online sexually explicit materials, future studies focusing on identifying risk factors for individuals (particularly youth) at risk for developing CSB are warranted.

## Supporting Information

File S1 **Supporting information.**
(DOCX)
