# Supplementary material for: Neural Correlates of Sexual Cue Reactivity in Individuals with and without Compulsive Sexual Behaviours
Source: PLoS One. 2014 Jul 11;9(7):e102419. doi: 10.1371/journal.pone.0102419 (PMC4094516; doi:10.1371/journal.pone.0102419)
Supplement: File S1 — Supporting information. (DOCX) [file pone.0102419.s001.docx]

**Supplementary materials**

Methods

A modified version of the Arizona Sexual Experiences Scale (ASES) [[1](#_ENREF_1)] was used with one version relevant to intimate relationships and another version relevant to online sexually explicit material. Questions included: ‘Q1. How strong is your sex drive?’ ‘Q2. How are you sexually aroused (turned on)?’ ‘Q3. Can you easily get and keep an erection?’ Questions 4 and 5 were relevant to sexual activity or pornography use in the last week. ‘Q4. How easily can you reach an orgasm?’ and ‘Q5. Are your orgasms satisfying?’ Questions were answered on a scale of 1 (e.g. extremely strong; extremely easily) to 8 (e.g. no sex drive; never), with higher scores representing greater subjective impairment.

Imaging acquistion

T2*-weighted echo-planar images (EPI) were acquired with blood oxygenation level-dependent (BOLD) contrast on a 3.0 Tesla magnetic resonance scanner (Trio, Siemens) with a 32-channel head coil using a tilted plane acquisition at the Wolfson Brain Imaging Centre. Thirty-nine interleaved slices were acquired (TR 2.32 s, TE 33 ms, 3 mm slice thickness, no gap). T1-weighted structural images (TR=2300 ms; TE=2.98 ms; FOV 240 x 256 x 176 mm, voxel size 1x1x1 mm) were acquired.

The first 5 volumes of each session were discarded to allow for T1 equilibration effects. Pre-processing consisted of slice-timing correction, spatial realignment, co-registration with the T1-weighted structural image, normalization, and spatial smoothing using a Gaussian kernel with a full-width at half-maximum of 8 mm. To correct for motion artefacts, subject-specific realignment parameters were included in the GLM. Imaging data were analyzed using Statistical Parametric Mapping software (SPM8) (<http://www.fil.ion.ucl.ac.uk/spm>).

References

1. McGahuey CA, Gelenberg AJ, Laukes CA, Moreno FA, Delgado PL, et al. (2000) The Arizona Sexual Experience Scale (ASEX): reliability and validity. J Sex Marital Ther 26: 25-40.

2. Carnes P, Delmonico DL, Griffin E (2001) In the Shadows of the Net: Breaking Free from Compulsive Online Sexual Behaviour, 2nd Ed. Center City, Minnesota: Hazelden

3. Kafka MP (2010) Hypersexual disorder: a proposed diagnosis for DSM-V. Arch Sex Behav 39: 377-400.

4. Reid RC, Carpenter BN, Hook JN, Garos S, Manning JC, et al. (2012) Report of findings in a DSM-5 field trial for hypersexual disorder. J Sex Med 9: 2868-2877.

Table S1. Diagnostic criteria for compulsive sexual behaviour

|  | Diagnostic criteria |
| --- | --- |
| Sexual addiction [[2](#_ENREF_2)] | Three or more of the following criteria:   1. Recurrent failure to resist impulses 2. Frequently engaging to a greater extent or over a longer period 3. Persistent desire or unsuccessful effots to stop, reduce or control 4. Inordinate amount of time spent 5. Preoccupation 6. Frequently engaging in behavior when expected to fulfill occupational, academic, domestic or social obligations 7. Continuation of behaviour despite knowledge of persistent social, academic, financial or physical problem 8. Increase intensity, frequency, number or risk of behavior to achieve desired effect of diminished effect of continued behavior 9. Giving up or limiting social, occupational or recreational activities 10. Distress, anxiety or restlessness if unable to engage in behaviour |
| DSM-5 proposed criteria for hypersexual disorder [[3](#_ENREF_3),[4](#_ENREF_4)] | 1. Recurrent and intense sexual fantasies urges or behaviours associated with 4 or more of the following criteria: 2. Excessive time spent 3. Repetitively engaging in response to dysphoric mood states 4. Repetitively engaging in response to stressful life events 5. Repetitive but unsuccessful attempts to control or reduce 6. Repetitively engaging while disregarding risk for physical or emotional harm to self or others 7. Clinically significant personal distress or impairment in social, occupational or other important areas of functioning associated with frequency and intensity of sexual fantasies, urges or behaviours 8. Not due to physiological effects of exogenous substances, medical condition or manic episode 9. 18 years or older   Specify if: masturbation, pornography, sexual behavior with consenting adults, cybersex, telephone sex and strip clubs |

Table S2. Subject characteristics

Abbreviations: CSB = subjects with compulsive sexual behavior; HV = healthy volunteers; BES = Binge Eating Scale; AUDIT = Alcohol Use Disorders Identification Test; BDI = Beck Depression Inventory; SSAI/STAI = Speilberger State and Trait Anxiety Inventory; OCI-R = Obsessive Compulsive Inventory; UPPS-P = UPPS Impulsive Behaviour Scale

|  |  | CSB | HV | T | P |
| --- | --- | --- | --- | --- | --- |
| Number |  | 19 | 19 |  |  |
| Abstinence (days) |  | 27 (25.72) |  |  |  |
| Education | High school  Current Univ. student  College degrees  Univ. undergraduate degree  Masters degree | 19  5  3  8  5 | 19  7  4  4  1 |  |  |
| IQ |  | 111.27 (5.39) | 110.96 (7.95) | 0.141 | 0.889 |
| Relationship status | Single  Current relationship  Married | 9  6  4 | 8  8  3 |  |  |
| Occupation | Student  Part-time work  Full-time work  Unemployed | 5  3  11  0 | 7  0  10  2 |  |  |
| Medications | Antidepressants | 2 |  |  |  |
| Body mass index |  | 24.35 (3.55) | 23.09 (4.13) | 1.009 | 0.320 |
| Binge Eating | BES | 7.50 (6.42) | 5.90 (6.60) | 0.758 | 0454 |
| Alcohol use | AUDIT | 7.51 (4.29) | 6.75 (3.56) | 0.594 | 0.556 |
| Depression | BDI | 12.44 (9.34) | 4.19 (4.75) | 3.432 | 0.002 |
| Anxiety | SSAI | 44.10 (13.63) | 36.52 (13.04) | 1.752 | 0.089 |
|  | STAI | 49.47 (13.25) | 37.13 (14.82) | 2.706 | 0.010 |
| Obsessive compulsive | OCI-R | 19.33 (11.16) | 12.36 (11.74) | 1.876 | 0.069 |
| Impulsivity | UPPS-P | 151.05 (17.27) | 129.57 (22.53) | 3.298 | 0.002 |

Table S3. Behavioural ratings

Abbreviations: CSB = subjects with compulsive sexual behavior; HV = healthy volunteers; ISST = Internet Sex Screening Test; ASES = Arizona Sexual Experiences Scale; YIAT = Young Internet Addiction Test; CIUS = Compulsive Internet Use Scale

|  |  | CSB | HV | T or F | P |
| --- | --- | --- | --- | --- | --- |
| ISST |  | 15.95 (3.96) |  |  |  |
| ASES: relationship | -Sex drive  -Sexually aroused  -Erection  -Reaching orgasm  -Satisfaction of orgasm | 3.11 (1.78)  3.33 (1.41)  3.00 (1.49)  2.33 (1.11)  2.33 (0.50) | 2.76 (0.62)  2.62 (0.49)  2.19 (0.68)  2.38 (0.65)  2.15 (0.90) | 0.71  4.70  4.98  0.02  0.29 | 0.405  0.037  0.032  0.893  0.594 |
| ASES: pornography | -Sex drive  -Sexually aroused  -Erection  -Reaching orgasm  -Satisfaction of orgasm | 3.11 (1.44)  2.89 (1.19)  2.68 (1.20)  1.83 (0.58)  2.75 (0.87) | 2.89 (0.74)  2.84 (0.60)  2.32 (0.82)  2.50 (0.65)  2.43 (0.94) | 0.32  0.03  1.22  7.52  0.81 | 0.576  0.865  0.278  0.011  0.376 |
| Internet use | YIAT | 53.21 (14.79) | 19.38 (13.02) | 7.484 | <0.001 |
|  | CIUS | 38.05 (8.62) | 9.67 (6.98) | 11.153 | <0.001 |

Table S4. Main imaging effects of condition

Abbreviations: R = right; L = left; cx = cortex; OFC = orbitofrontal cortex; sup = superior

| Contrast | Region |  | x y z (mm) | Z | Cluster | P |
| --- | --- | --- | --- | --- | --- | --- |
| Explicit - Exciting | Occipito-temporal cx | R  L | 42 -68 -10  -46 -72 -8 | Inf  Inf | 8850 | <0.0001  <0.0001 |
|  | L Parietal cx | L supramarginal  L inferior | -62 -24 28  -28 -54 54 | Inf  7.60 | 2045 | <0.0001  <0.0001 |
|  | R Parietal cx | R superior | 30 -52 56 | Inf | 966 | <0.0001 |
|  | Insula/cingulate/ OFC | Bilat ant insula/ ant cing / lateral OFC / VS/ pallidum/ hypothal/ amygdala / SN | -40 10 -4  0 34 10 | Inf  7.45 | 9092 | <0.0001  <0.0001 |
|  | R parietal | Postcentral | 62 -18 34 | 6.60 | 508 | <0.0001 |
|  | Caudate | R | 18 0 24 | 6.22 | 211 | <0.0001 |
|  | Frontal | R precentral | 56 12 38 | 5.76 | 245 | <0.0001 |
|  | Insula | L posterior insula | 38 2 14 | 5.30 | 21 | 0.001 |
|  | Cingulate | Mid cingulate | 0 -14 36 | 5.27 | 30 | 0.002 |
|  | Cerebellum |  | -2 -54 -30 | 5.22 | 51 | 0.003 |
|  | Frontal | R Inferior PFC | 54 44 10 | 5.20 | 35 | 0.003 |
| Erotic - Exciting | R occipito-temporal/ R sup parietal/ cerebellum |  | 52 -60 -8  30 -56 60  -8 -78 -34 | Inf | 121230 | <0.0001 |
|  | R inferior frontal |  | 52 14 32 | 6.90 | 1676 | <0.0001 |
|  | L sup parietal |  | -26 -60 56 | 7.17 | 818 | <0.0001 |
|  | L inferior frontal |  | -40 2 28 | 5.89 | 810 | <0.0001 |
|  | R caudate |  | 12 12 2 | 5.43 | 90 | 0.003 |
| Money - Exciting | R parietal | Postcentral | 48 -30 46 | 6.49 | 383 | <0.0001 |
|  | R parietal | Angular gyrus | 30 -56 44 | 6.11 | 740 | <0.0001 |
|  | L superior parietal |  | -34 -64 58 | 5.45 | 333 | 0.001 |
|  | L inferior frontal |  | -50 12 26 | 5.42 | 381 | 0.001 |
|  | R occipital |  | 26 -98 10 | 5.32 | 69 | 0.002 |
|  | R inferior frontal |  | 58 12 36 | 4.99 | 71 | 0.009 |
|  | L inferior frontal |  | -46 36 14 | 4.97 | 108 | 0.009 |

Table S5. Main effects of condition focusing on hypothesized regions of interest

Abbreviations: dACC = dorsal anterior cingulate

| Contrast | Region |  | x y z (mm) | Z | P |
| --- | --- | --- | --- | --- | --- |
| Explicit – Exciting | Ventral striatum | L  R | -22 14 -18  22 2 -16 | 6.21  5.72 | <0.0001  <0.0001 |
|  | dACC |  | 0 26 28 | 7.33 | <0.0001 |
|  | Pallidum | L  R | -10 2 0  20 -4 -6 | 5.64  5.46 | <0.0001  <0.0001 |
|  | Amygdala | R  L | 22 -4 -12  -18 -4 -14 | 7.29  6.53 | <0.0001  <0.0001 |
|  | Substantia nigra | R  L | 8 -12 -10  -12 -22 -12 | 5.49  5.09 | <0.0001  <0.0001 |
|  | Hypothalamus | R | 4 -6 -10  -8 -6 -8 | 6.38  6.02 | <0.0001  <0.0001 |
|  |  |  |  |  |  |
| Explicit – Erotic | Ventral striatum | R  L | 10 22 -8  -8 22 -10 | 3.83  3.90 | 0.020  0.008 |
|  | dACC |  | -2 22 29 | 4.59 | 0.002 |
|  | Amygdala | R  L | 22 -8 -12  -18 -4 -12 | 4.11  3.34 | 0.001  0.008 |
|  | Hypothalamus | R  L | 4 -2 10  -4 -4 -8 | 3.82  2.83 | 0.002  0.023 |
